# Endogenous calcitonin gene-related peptide in cerebrospinal fluid and early quality of life and mental health after good-grade spontaneous subarachnoid hemorrhage—a feasibility series

**DOI:** 10.1007/s10143-020-01333-z

**Published:** 2020-06-22

**Authors:** Elisabeth Bründl, Martin Proescholdt, Eva-Maria Störr, Petra Schödel, Sylvia Bele, Julius Höhne, Florian Zeman, Alexander Brawanski, Karl-Michael Schebesch

**Affiliations:** 1 Department of Neurosurgery, University Medical Center Regensburg, Franz-Josef-Strauß-Allee 11, 93053 Regensburg, Germany; 2 Center for Clinical Studies, University Medical Center Regensburg, Franz-Josef-Strauß-Allee 11, 93053 Regensburg, Germany

**Keywords:** Calcitonin gene-related peptide, CGRP, Health-related quality of life, Impairment, Neuropsychological outcome, Spontaneous subarachnoid hemorrhage

## Abstract

The vasodilatory calcitonin gene-related peptide (CGRP) is excessively released after spontaneous subarachnoid hemorrhage (sSAH) and modulates psycho-behavioral function. In this pilot study, we prospectively analyzed the treatment-specific differences in the secretion of endogenous CGRP into cerebrospinal fluid (CSF) during the acute stage after good-grade sSAH and its impact on self-reported health-related quality of life (hrQoL). Twenty-six consecutive patients (f:m = 13:8; mean age 50.6 years) with good-grade sSAH were enrolled (drop out 19% (*n* = 5)): 35% (*n* = 9) underwent endovascular aneurysm occlusion, 23% (*n* = 6) microsurgery, and 23% (*n* = 6) of the patients with perimesencephalic SAH received standardized intensive medical care. An external ventricular drain was inserted within 72 h after the onset of bleeding. CSF was drawn daily from day 1–10. CGRP levels were determined via competitive enzyme immunoassay and calculated as “area under the curve” (AUC). All patients underwent a hrQoL self-report assessment (36-Item Short Form Health Survey (SF-36), ICD-10-Symptom-Rating questionnaire (ISR)) after the onset of sSAH (t_1_: day 11–35) and at the 6-month follow-up (t_2_). AUC CGRP (total mean ± SD, 5.7 ± 1.8 ng/ml/24 h) was excessively released into CSF after sSAH. AUC CGRP levels did not differ significantly when dichotomizing the aSAH (5.63 ± 1.77) and pSAH group (5.68 ± 2.08). aSAH patients revealed a higher symptom burden in the ISR supplementary item score (*p* = 0.021). Multiple logistic regression analyses corroborated increased mean levels of AUC CGRP in CSF at t_1_ as an independent prognostic factor for a significantly higher symptom burden in most ISR scores (compulsive-obsessive syndrome (OR 5.741, *p* = 0.018), anxiety (OR 7.748, *p* = 0.021), depression (OR 2.740, *p* = 0.005), the supplementary items (OR 2.392, *p* = 0.004)) and for a poorer performance in the SF-36 physical component summary score (OR 0.177, *p* = 0.001). In contrast, at t_2_, CSF AUC CGRP concentrations no longer correlated with hrQoL. To the best of our knowledge, this study is the first to correlate the levels of endogenous CSF CGRP with hrQoL outcome in good-grade sSAH patients. Excessive CGRP release into CSF may have a negative short-term impact on hrQoL and emotional health like anxiety and depression. While subacutely after sSAH, higher CSF levels of the vasodilator CGRP are supposed to be protective against vasospasm-associated cerebral ischemia, from a psychopathological point of view, our results suggest an involvement of CSF CGRP in the dysregulation of higher integrated behavior.

## Introduction

Spontaneous subarachnoid hemorrhage (sSAH) represents a complex and still devastating neurovascular disease, associated with substantial morbidity and mortality. Over the past 30 years, advances in neurovascular treatment strategies and specialized neurocritical care have led to decreasing case fatality rates [1, 2] with an absolute annual reduction in 30-day mortality of 0.9% over the past decades [1]. However, the mortality in sSAH patients is still as high as 50% [3]. The in-hospital mortality is estimated 18% [4]. About 85% of the non-traumatic spontaneous events comprise aneurysmal subarachnoid hemorrhages (aSAH) and 10% are non-aneurysmal perimesencephalic subarachnoid hemorrhages (pSAH) [5]. Epidemiologically, aSAH and pSAH are two diseases with different evolutions [6]. Compared with aSAH, pSAH represents a subarachnoid hemorrhage (SAH) entity with a very distinct and usually more benign clinical course [6–8]. The survivors harbor serious risks of neurological dysfunction, functional disability, and cognitive impairment [9, 10], even months to years after ictus [11, 12]. There is a marked disparity between reattained functional independence in up to 70% [13] of the sSAH patients and considerable long-term neuropsychological deficits [9, 10, 14] in up to 94.6% [15] with a reduced health-related quality of life (hrQoL) in 35% of the patients 1 year after sSAH [12, 16], anxiety (in up to 54%) [11, 17], depression (approaching 61.7%) [17, 18], and, in up to two-thirds [9, 19], the inability to reassume one’s previous occupation [19, 20] [9, 14].

The underlying pathomechanisms are poorly understood and deemed to be multifactorially mediated [9, 21, 22]. A combination of focal and diffuse brain injury is assumed, probably due to the primary insult, determined by the severity of the bleeding, or subsequent profound secondary complications, most notably the arterial cerebral vasospasm (CV) and the delayed cerebral ischemia [23]. In light of this, the early identification of reliable predictive outcome parameters in sSAH is as tempting as challenging. When evaluating the potential pathophysiological role of vasoactive endogenous neuropeptides in sSAH-related cerebral hemodynamic changes, CV-induced cerebral ischemia, and outcome after sSAH, neuropeptide Y (NPY) [24–27] and calcitonin gene-related peptide (CGRP) [28–33] have gained paramount interest.

The 37-amino acid neuropeptide CGRP, firstly described in 1982 [34], is a highly potent microvascular vasodilator [35] and neuromodulator [36], which is widely expressed and stored in the central and peripheral nervous system [37]. In the cerebral circulation, CGRP is released from presynaptic vesicles in sensory perivascular fibers that almost exclusively originate from the gasserian ganglion [38, 39]. Physiologically, together with other neuropeptides, CGRP restores the cerebrovascular tone in response to vasoconstriction via a remarkable relaxation of the smooth muscle layer, hereby dilating the arteries and, consecutively, increasing the cerebral blood flow (CBF) [29]. Through this dynamic reflex, termed the “trigemino-vascular response”, CGRP opposes excessive vasoconstriction [39]. In aSAH, CGRP has been demonstrated to be excessively released into cerebrospinal fluid (CSF) [31, 33] with a potential neuroprotective effect by preventing CV and cerebral ischemia [33].

Besides its eminent vasoactive role, peptidergic psychoactive implications of CGRP have been repeatedly described in humans and, translationally, in various animal models, with a crucial involvement in multifaceted neurobehavioral processes [40] such as depression [41–47], anxiety [48], learning and memory [49], possibly in dementia [50], in the pathophysiology of inflammatory and neuropathic pain [51–53], and, by unalterable cerebral vasodilation, in migraine [54–56]. To date, the behavioral profile of the action of supraspinal CGRP has insufficiently been elucidated, though.

To the best of our knowledge, no data are yet available on the relevance of CGRP in supratentorial CSF on hrQoL outcome after sSAH in humans. We hypothesize that the excessive release of endogenous CGRP in the subacute phase after sSAH might impact quality of life, even in good-grade patients.

## Patients and methods

### Ethical approval

All procedures performed in studies involving human participants (i.e. the clinical database, the prospective liquid biobanking, and the study protocol) were in accordance with the ethical standards of the institutional research committee (Ethikkommission des Universitätsklinikums Regensburg, Ethikvotum 06-179) and with the 1964 Helsinki declaration and its later amendments or comparable ethical standards.

### Patient population

The cohort has been reported previously [21, 24]. Twenty-six consecutive patients with acute non-traumatic, angiographically confirmed aneurysmal or non-aneurysmal sSAH in prognostically favorable neurological condition were prospectively enrolled in this single-center trial at our University Medical Center between February 2013 and May 2016.

#### Study selection criteria

After obtaining written informed consent, we selectively included native German speakers, aged 18 to 75 years, with non-traumatic sSAH. The patients either underwent microsurgical aneurysm occlusion (MS group) or endovascular aneurysm occlusion (EV group) for an intracranial aneurysm in the anterior or posterior circulation (aSAH). Patients with a non-aneurysmal pSAH received standardized treatment in the intensive care unit (ICU) (pSAH group). Each patient had been admitted to hospital within 48 h of ictus in prognostically favorable, good to moderate neurological condition, that means with a Hunt and Hess (HH) score [57] of 1 to 4 or a World Federation of Neurosurgical Societies (WFNS) score [58] of 1 to 4 and an initial Glasgow Coma Scale (GCS) of ≥ 9. Within the first 72 h after the onset of sSAH, all patients received an external ventricular drain because of a radiologically confirmed acute occlusive hydrocephalus. Exclusion criteria were (1) preceding neurosurgical or neurovascular procedures, (2) a previous history of intracranial disorders, (3) a previous history of psychiatric or neurodegenerative diseases, (4) severe autoimmune or systemic diseases, (5) (giant) aneurysm causing mass effect, and (6) severe postprocedural complications, such as intracranial bleeding after treatment of aneurysm or clinically symptomatic cerebral ischemia.

The clinical database comprised all demographic, neurological, and radiological variables, comorbidities, non-/invasive procedures, complications, outcome grading (Glasgow Outcome Scale [GOS] [59] and modified Ranking Scale [mRS] [60]), and comprehensive pharmacological screening (at discharge and at the 6-month FU). All patients were examined by means of cerebral computed tomography (CT) and digital subtraction angiography (DSA) and treated according to our ICU standard operating protocol [61]. Transcranial Doppler ultrasound examinations [62] were conducted daily. Our neuroradiologists individually decided on the timing and number of DSA controls on a patient-to-patient basis, depending on the initial DSA findings.

#### Therapeutic procedures

For the aSAH patients, neurosurgeons and neuroradiologists decided on the treatment modality after interdisciplinary consent. Our standardized surgical and endovascular procedure protocols have been described elsewhere [63].

### Self-reported assessment of hrQoL and mental health

Outcome evaluation was conducted in a single session in a noisefree setting by having the participants complete both surveys, as an inpatient at t_1_ and as an outpatient at t_2_, respectively. No effects of fatigue were apparent. FU assessment additionally comprised a neurological examination and a non-standardized semi-structured interview, including the patient’s subjective health status, the current medication, and the employment status. All patients completed the German version of the 36-Item Short Form Health Survey (SF-36) [64] (a performance score) and the ICD-10-Symptom-Rating questionnaire (ISR) [65] (a score for symptom burden) in the subacute phase after the onset of bleeding (between day 11 and 35 after sSAH; t_1_) and in the short term (chronic phase) at the 6-month FU (t_2_).

#### ISR

The ISR aims at a comprehensive evaluation of the severity of psychological disorders. The ISR 2.0 comprises 29 items and six syndrome scales: depression, anxiety, obsessive/compulsive disorders, somatoform disorders, eating disorders, and a supplementary scale, which covers a variety of syndromes (including concentration, suicidality, sleep, appetite, obliviousness, flash backs, problems with activities of daily living, feelings of displacement and alienation, non-organic sexual dysfunction), as well as a total score. Each syndrome scale ranges from a minimum of 0 (best performance) to a maximum of 4 points with higher scores indicating a more severe symptom burden. Cutoff values for each syndrome scale grade the degree of severity of symptoms in “suspected”, “mild”, “moderate”, and “severe” [65].

#### SF-36

The SF-36 is a 36-item generic general health questionnaire that yields scores on eight health subscales relating to physical health (physical functioning (Pfi), role limitations due to physical health problems (Rolph), bodily pain (Pain), general health perceptions (Ghp)) and psychological health (vitality (Vital), social functioning (Social), role limitations because of emotional problems (Rolem), and general mental health (Mhi)). These eight subscales can be summarized in a corresponding physical component summary (PCS) and a mental component summary (MCS). The SF-36 also includes a single item that provides an indication of perceived change in health (health transition item, Rawhtran). Each item is scored on a 0 to 100 range and a high score defines a more favorable health state. Items in the same scale are averaged together to create the 8 scale scores [64, 66].

### Laboratory procedures

CSF was drawn directly from the external ventricular drain and collected daily over the 10-day period after the onset of sSAH. Immediately after sampling, the samples were centrifuged at 1200 rpm for 10 min, and the supernatants were aliquoted and stored at − 80 °C until further use. The samples were thawed, aliquoted (1 ml), evaporated on a vacuum concentrator (Christ RVC 2-25 CD plus; Osterode am Harz, Germany), and dissolved in 250 μl of buffer resulting in a fourfold concentration. CGRP levels were measured in duplicate CSF samples using competitive enzyme immunoassay (EIA; Phoenix Pharmaceuticals Inc., Burlingame, CA). According to pharmacological studies, cerebral exposure to the released endogenous CSF CGRP over time was measured as area under the curve (AUC) and expressed as ng/ml × 24 h.

### Statistical analysis

Continuous data and test results on hrQoL and mental health are presented as mean ± standard deviation (SD) and range (minimum to maximum) and categorical data as frequency counts.

hrQoL assessment: Changes over time within each group were analyzed with a paired *t* test. Differences between groups at postinterventional assessment were analyzed with an analysis of variance (ANOVA) followed by Fisher’s LSD post hoc pairwise comparisons.

Correlation of CGRP with hrQoL assessment: Univariate and multiple logistic regression analyses were conducted for correlations of AUC CGRP with hrQoL test scores and/or clinical variables. Changes over time within each group were analyzed with a paired *t* test. Intragroup variances (correlations between hrQoL test scores and clinical variables) were analyzed using an analysis of variance (Bartlett’s test for equal variances). Statistical analysis was conducted according to Stata procedures (Stata Version 14.2; Stata Corp. College Station, TX, USA).

A *p* value of < 0.05 was considered statistically significant.

## Results

### Demographics and descriptive statistics

**Fig. 1:**
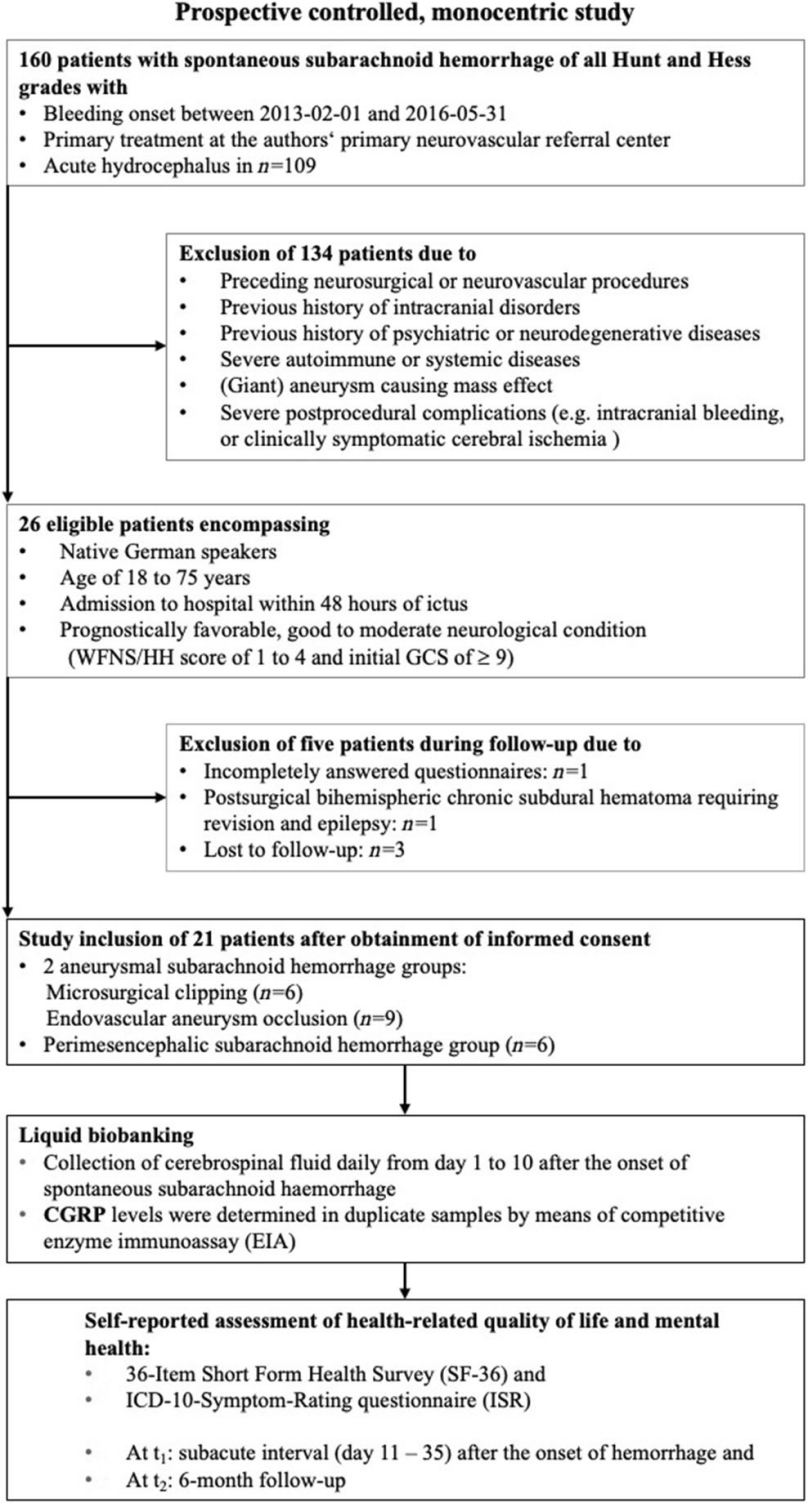
**Flowchart:** Study selection criteria, reasons for exclusion of potentially eligible patients, and study design in terms of liquid biobanking and health-related quality of life assessment

During February 2013 and May 2016, a total of 160 sSAH patients of all HH grades was admitted and treated in our medium volume neurovascular center. Among these, 109 patients presented with an acute hydrocephalus requiring CSF drainage via external ventricular drain. Applying our strictly defined selection criteria, only 26 good-grade sSAH patients could initially be enrolled. Another five patients had to be excluded from analysis during FU (lost to FU: *n* = 3; incompletely answered questionnaires: *n* = 1; postsurgical bihemispheric chronic subdural hematoma requiring revision and epilepsy: *n* = 1). Accordingly, 21 consecutive patients with good-grade sSAH (8 men, 13 women) were included as depicted in the flowchart (see Fig. 1). Mean age was 50.6 years (range 27 to 72 years). Our cohort encompassed three patients (14%) with HH grade III and even one patient (5%) with HH grade IV. As all of these four aSAH patients neurologically improved immediately after insertion of an external ventricular drain following hospital admission (i.e., HH I or HH II), the poorer HH score was obviously related to acute occlusive hydrocephalus. With this qualification, we consider the term “good-grade” sSAH patients as appropriate for our cohort. Surprisingly, our good-grade sSAH population exclusively encompassed patients with severe radiological grades of sSAH (Fisher score 3 or 4) with concomitant hydrocephalus. Consecutively, the presented cohort is imbalanced in terms of the overestimation of the true hydrocephalus rate after sSAH. Statistical intergroup comparisons yielded no significant differences except for a higher number of middle cerebral artery aneurysms in the MS group (*n* = 3, EV *n* = 0, pSAH *n* = 1 (unruptured aneurysm); *p* = 0.022), a higher intake of antiplatelets in the EV group (*n* = 5, MS and pSAH *n* = 0 each; *p* = 0.016) at t_1_, an unsurprisingly longer duration (mean ± SD) of MS vs. EV (MS 242.33 ± 24.75 min (range 205–279) vs. EV 136.67 ± 46.87 min (range 55–200); *p* = 0.004), and a longer mean time (mean ± SD) spent on mechanical ventilatory support in the EV group than in the pSAH group (EV 117.99 ± 264.14 h (range 6.92–808.83); pSAH 5.82 ± 6.34 h (range 0–14.83); MS 116.03 ± 244.14 h (range 2.17–613.63); EV vs. pSAH *p* = 0.0496; EV vs. MS *p* = 0.864, MS vs. pSAH *p* = 0.065). The baseline data including the aneurysm site, GCS, HH, WFNS and Fisher score, procedure variables, medication, and outcome grading has previously been reported [21, 24].

### Neuropsychological assessment

The self-reported performance in hrQoL and mental health has been reported previously [21, 24]. We conducted intragroup comparisons and variance analyses: (1) Within 6 months (from t_1_ to t_2_), sSAH patients had significantly improved with regard to depression, anxiety, Pfi, Pain, Ghp, Social, and in the PCS. (2) When dichotomizing aSAH versus pSAH patients, no significant differences in terms of SF-36 and ISR scores were detected either except for a significantly higher symptom burden for aSAH patients in the ISR supplementary items score (*p* = 0.021). (3) Poor self-reported hrQoL performance (ISR scores: total, depression, compulsive-obsessive; physical SF-36 items: Rolph, Pain, Ghp) in the subacute phase correlated with worse outcome on the GOS at discharge. (4) Neurological status at hospital admission in terms of the HH score correlated positively with all psychological SF-36 item scores at t_1_ (Vital, Social, Rolem, Mhi, MCS). Univariate regression analysis did not yield any significant correlations between cognitive outcome and further clinical variables such as GCS, WFNS, and the Fisher score. However, multivariate analysis revealed various significant correlations between the respective hrQoL domains and clinical variables (compulsive-obsessive syndrome vs. treatment modality (*p* = 0.008); anxiety vs. Fisher score (*p* = 0.040), anxiety vs. age (*p* = 0.045), anxiety vs. gender (*p* = 0.031); depression vs. WFNS grade (*p* = 0.027), depression vs. GCS (*p* = 0.009), depression vs. treatment modality (*p* = 0.023), depression vs. TCD-based CV (*p* = 0.011); supplementary items vs. treatment modality (*p* = 0.026); Ghp vs. treatment modality (*p* = 0.003), Ghp vs. TCD-based CV (*p* = 0.008); PCS vs. HH grade (*p* = 0.017), PCS vs. Fisher grade (*p* = 0.038), PCS vs. GCS (*p* = 0.042), PCS vs. age (*p* = 0.026), and PCS vs. gender (*p* = 0.007)).

Pearson’s correlation analysis revealed significant correlations between all hrQoL domains except for (1) somatoform syndrome vs. depression, (2) nutrition disorder vs. depression, (3) nutrition disorder vs. compulsive/obsessive syndrome, (4) nutrition disorder vs. somatoform syndrome, and (5) supplementary items score vs. nutrition disorder.

### Correlation of neuropsychological performance with CGRP exposure in CSF

AUC CGRP (total 5.7 ± 1.8 ng/ml/24 h) was excessively released into CSF after sSAH. Mean CGRP levels ranged highest in the EV group (6.1 ± 2.1 ng/ml/24 h) followed by the pSAH group (5.7 ± 2.1 ng/ml/24 h), and the MS group (5.0 ± 1.1 ng/ml/24 h). The mean total AUC CGRP level in CSF averaged 5.7 ± 1.8 ng/ml/24 h. The AUC CGRP levels were separately calculated for aSAH (5.63 ± 1.77 ng/ml/24 h) and pSAH (5.68 ± 2.08 ng/ml/24 h) and did not differ significantly (*p* = 0.521). The analysis of the distinct AUC curves over the first 10 days revealed a highly interindividual pattern without consistency of the AUC dynamics.

**Fig. 2:**
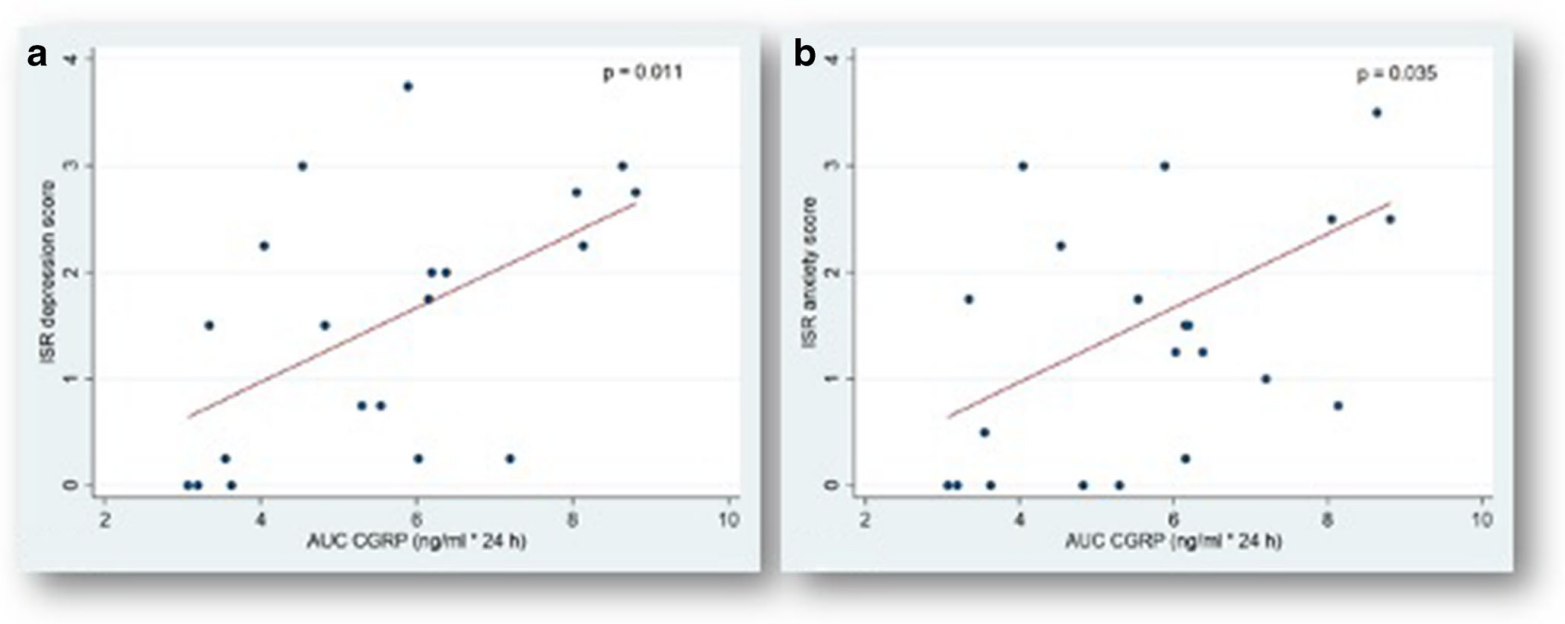
*Correlation of elevated area under the curve (AUC) values of calcitonin gene-related peptide (CGRP) in cerebrospinal fluid (CSF) with a higher symptom burden in the ICD-10-Symptom-Rating questionnaire (ISR) scores within the first 10 days after the onset of spontaneous subarachnoid hemorrhage.* Area under the curve (AUC) values of endogenous calcitonin gene-related peptide (CGRP) in supratentorial cerebrospinal fluid (CSF) within the first 10 days after the onset of spontaneous subarachnoid hemorrhage plotted versus the ICD-10-Symptom-Rating questionnaire (ISR) scores [65] in **a** depression and **b** anxiety. The ISR with 29 items and 6 syndrome scales aims at comprehensively evaluating the severity of psychological disorders. Each syndrome scale ranges from a minimum of 0 (best performance) to a maximum of 4 points with higher scores indicating a more severe symptom burden. Each dot represents the mean level of CSF CGRP in [ng/ml × 240 h] for each patient, indicating a significant linear correlation (compare regression line) with a higher symptom burden. *p < 0.05

**Fig. 3:**
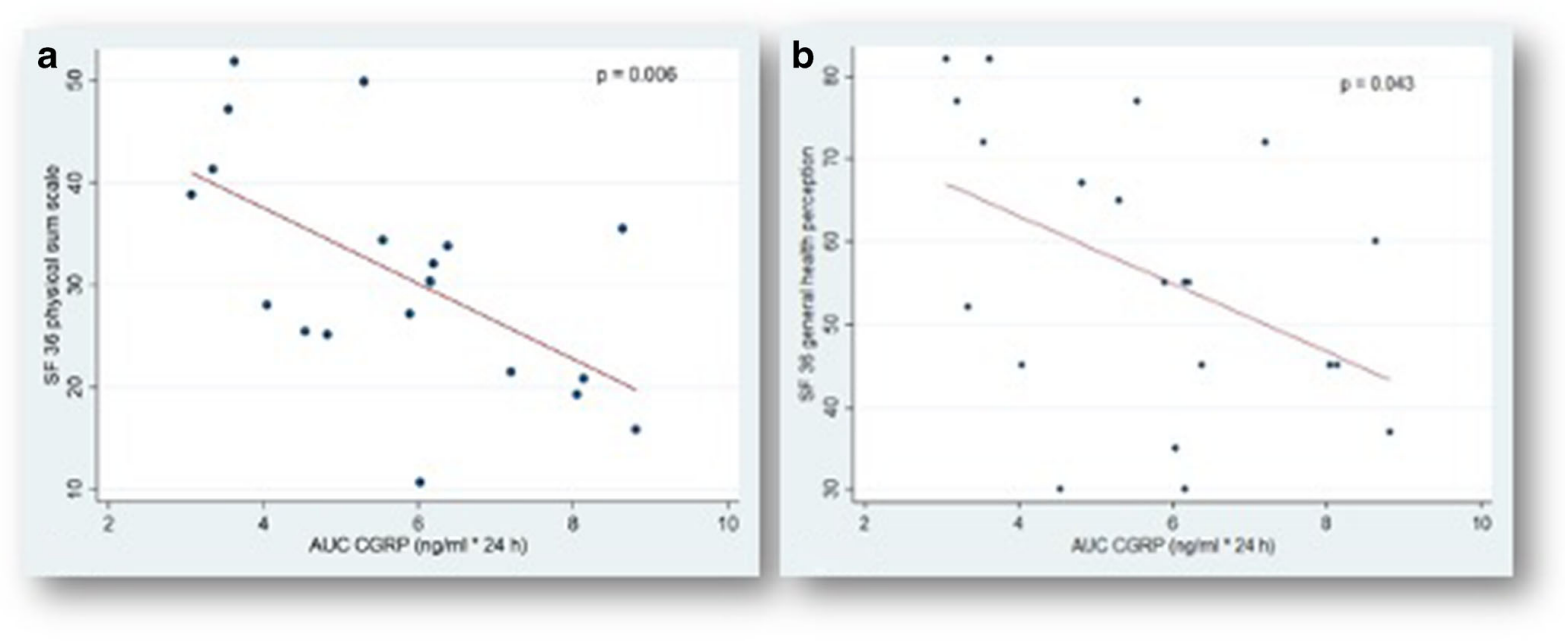
*Correlation of the area under the curve (AUC) values of calcitonin gene-related peptide (CGRP) in cerebrospinal fluid (CSF) with reduced general health perception and an impaired physical component summary score in the 36-Item Short Form Health Survey (SF-36) within the first 10 days after the onset of spontaneous subarachnoid hemorrhage.* Area under the curve (AUC) values of endogenous calcitonin gene-related peptide (CGRP) in supratentorial cerebrospinal fluid (CSF) within the first 10 days after the onset of spontaneous subarachnoid hemorrhage plotted versus the 36-Item Short Form Health Survey (SF-36) [64] scores in **a** the physical component summary score (PCS) and in **b** general health perceptions (Ghp). The SF-36 is a 36-item generic general health questionnaire yielding scores on 8 health subscales relating to physical and psychological health. These 8 subscales can be summarized in a corresponding physical component summary and an MCS. Each item is scored in the range 0 to 100, and a high score defines a more favorable state of health. Items in the same scale are averaged together to create the 8 scale scores. Each dot represents the mean level of CSF CGRP in [ng/ml × 240 h] for each patient, indicating a significant linear correlation (compare regression line) with poorer performance in hrQoL and emotional health. *p < 0.05

Since AUC CGRP concentrations did not significantly differ between the subgroups, subsequent analyses were conducted for the whole study cohort (mean CSF CGRP AUC level 5.65 ± 1.81 ng/ml × 24 h; range 3.07 to 8.81 ng/ml × 24 h) without differentiation between the sSAH groups. The analysis of cognitive test performances and correlation with the AUC CGRP levels in CSF are summarized in Table 1. Increased mean values of AUC CGRP in CSF at t_1_ significantly correlated with a higher symptom burden in most ISR scores (compulsive-obsessive syndrome, anxiety, depression, somatoform syndrome, and in the supplementary items score) and with poorer performance in two physical SF-36 items (Ghp and the PCS). The respective regression analyses are depicted by means of scatterplots in Figs. 2a and b and 3a and b. In contrast, at the 6-month FU, CSF AUC CGRP concentrations over the first 10 days no longer showed any significant correlations with hrQoL test performance.

**Table 1:** Health-related quality of life and emotional health of the cohort (*n* = 21) and correlation with the calcitonin gene-related peptide concentrations in supratentorial cerebrospinal fluid in the subacute interval after the onset of spontaneous subarachnoid hemorrhage (t_1_) and at 6-month follow-up (t_2_)

Neuropsychological assessment	Test scores (mean ± SD)		Difference t_1_ vs. t_2_ (mean ± SD)	Paired *t* test t_1_ vs. t_2_ (*p* value)	AUC CGRP (*p* value)	
	t_1_	t_2_			t_1_	t_2_
ISR scores						
Depression	1.5 ± 1.2	1.1 ± 0.9	0.4 ± 1.1	0.046*	0.011*	0.431
Anxiety	1.3 ± 1.1	0.7 ± 0.9	0.7 ± 0.3	0.019*	0.035*	0.668
Compulsive-obsessive	0.8 ± 1.0	0.8 ± 1.0	0.0 ± 1.4	0.480	0.022*	0.678
Somatoform	0.6 ± 0.7	0.4 ± 0.6	0.1 ± 1.0	0.252	0.013*	0.438
Nutrition disorder	0.6 ± 1.0	0.6 ± 0.7	− 0.0 ± 1.2	0.523	0.421	0.898
Supplementary items	0.7 ± 0.5	0.5 ± 0.6	0.2 ± 0.6	0.064	0.015*	0.114
Total	0.9 ± 0.7	0.7 ± 0.6	0.2 ± 0.8	0.098	0.086	0.737
SF-36 scores						
Physical items						
Rawhtran	4.5 ± 0.6	2.9 ± 1.4	1.6 ± 1.4	1.000	0.066	0.499
Pfi	19.5 ± 28.9	72.4 ± 26.3	− 52.8 ± 35.5	0.001*	0.057	0.080
Rolph	39.3 ± 43.7	47.6 ± 45.3	− 8.3 ± 64.4	0.280	0.090	0.166
Pain	48.5 ± 34.6	67.2 ± 29.9	− 18.7 ± 37.9	0.018*	0.251	0.229
Ghp	56.3 ± 16.8	74.1 ± 18.2	− 17.8 ± 19.0	0.001*	0.043*	0.283
Psychological items						
Vital	51.0 ± 19.7	51.2 ± 20.3	− 0.2 ± 24.4	0.482	0.536	0.162
Social	66.1 ± 26.0	80.4 ± 25.2	− 014.3 ± 28.6	0.017*	0.453	0.653
Rolem	61.7 ± 46.2	61.7 ± 45.0	− 0.0 ± 58.8	0.500	0.835	0.102
Mhi	61.1 ± 20.6	68.2 ± 21.7	− 7.0 ± 28.6	0.137	0.621	0.808
PCS	31.0 ± 11.0	46.0 ± 10.0	− 13.3 ± 12.7	0.001*	0.006*	0.083
MCS	49.1 ± 12.5	47.5 ± 10.7	1.4 ± 12.9	0.677	0.582	0.549

*SD*, standard deviation; *test t*_*1*_, test in the subacute phase after the onset of bleeding (between day 11 to 35 after subarachnoid hemorrhage); *test t*_*2*_, test in the short-term (chronic phase) after treatment at 6-month follow-up; *AUC*, area under the curve; *CGRP*, calcitonin gene-related peptide; *ISR*, ICD-10-Symptom-Rating questionnaire; *SF-36*, German version of the 36-Item Short Form Health Survey; *Rawhtran*, health transition item; *Pfi*, physical functioning; *Rolph*, role limitations because of physical health problems; *Pain*, bodily pain; *Ghp*, general health perceptions; *Vital*, vitality; *Social*, social functioning; *Rolem*, role limitations because of emotional problems; *Mhi*, general mental health; *PCS*, physical component summary; *MCS*, mental component summary

*Statistical significance *p* < 0.05

Regression analyses did not reveal any significant correlation between AUC CGRP levels and patient variables like age, gender, treatment modality (MS vs. EV vs. pSAH), site of the aneurysm, HH grade, WFNS grade, Fisher grade, initial GCS, GOS at follow-up, and CV.

After having detected significant correlations between increased AUC CGRP levels and various hrQoL domains in the univariate analysis, we developed a multivariate model including AUC CGRP, age, gender, treatment modality (MS vs. EV vs. pSAH), site of the aneurysm, HH grade, WFNS grade, Fisher grade, initial GCS, and CV to analyze their impact on the respective hrQoL domains. As for the ISR, all factors that significantly correlated with the AUC CGRP in the univariate analysis were also found to be significant in the multivariate model except for somatoform syndrome (OR 83.991, *p* = 0.084). As for the SF-36, the PCS remained statistically significant, whereas the Ghp (OR 0.759, *p* = 0.282) were not (cf. Table 2). Conclusively, multiple logistic regression analyses corroborated AUC CGRP as an independent prognostic factor for outcome in terms of compulsive-obsessive syndrome (OR 5.741, *p* = 0.018), anxiety (OR 7.748, *p* = 0.021), depression (OR 2.740, *p* = 0.005), the supplementary items (OR 2.392, *p* = 0.004), and the PCS score (OR 0.177, *p* = 0.001).

**Table 2:** Multiple logistic regression analysis of health-related quality of life domains significantly correlated in the univariate analysis versus calcitonin gene-related peptide concentration in supratentorial cerebrospinal fluid in the subacute interval after the onset of spontaneous subarachnoid hemorrhage (t_1_)

hrQoL domains	Odds ratio	95% CI		*p* value
ISR				
Compulsive-obsessive syndrome	5.741	1.341	24.581	0.018*
Anxiety	7.748	1.366	43.959	0.021*
Depression	2.740	1.360	5.519	0.005*
Somatoform syndrome	83.991	.548	12,865.310	0.084
Supplementary items	2.392	1.328	4.310	0.004*
SF-36				
General health perceptions (Ghp)	0.759	0.459	1.255	0.282
Physical component summary (PCS)	0.177	0.065	0.481	0.001*

*hrQoL*, health-related quality of life; *test t*_*1*_, test in the subacute phase after the onset of bleeding (between day 11 to 35 after subarachnoid hemorrhage); *ISR*, ICD-10-Symptom-Rating questionnaire; *SF-36*, German version of the 36-Item Short Form Health Survey

*Statistical significance *p* < 0.05

## Discussion

Our prospective study advocated an association of increased CSF CGRP concentrations in the acute phase of sSAH with unfavorable short-term hrQoL.

For the past few decades, in sSAH research, neuroscientists and clinicians have increasingly focused on targeting novel molecular genetic, vascular, inflammatory, oxidative stress and protein biomarkers, especially in the CSF, to reliably predict functional, cognitive, and hrQoL outcomes for individualized treatment strategies [27, 67, 68]. Only one previous study [67] has addressed neurodegenerative ventricular CSF biomarkers in the context of poor hrQoL outcome after aSAH. Our research group [24, 27, 33] and others [31, 32, 68] have corroborated the predictive capacity of endogenous neuropeptides after sSAH, in particular, the vaso- and psychoactive CGRP.

### Calcitonin gene-related peptide propaedeutics

The multifunctional neuropeptide CGRP is an evolutionary, highly conserved 37 amino acid peptide that was firstly isolated from thyroid tissue in 1982 [34] and is derived from the calcitonin gene. CGRP exists in two isoforms, αCGRP and βCGRP. αCGRP, which is synthesized by alternative splicing of the calcitonin gene, represents the predominant form of CGRP within the body and is mainly expressed in the central and peripheral nervous system [34]. CGRP constitutes one of the most potent endogenous vasodilators in humans [32, 38]. In the cerebral circulation, CGRP is primarily stored in presynaptic vesicles of sensory fibers that are closely associated with blood vessels [69] and that almost exclusively arise from the trigeminal ganglia [39]. In response to the intrinsic release of vasoconstrictive neuropeptides, CGRP physiologically restores the vascular tone mediating the “trigeminovascular reflex” [29]. Beyond, literature has repeatedly highlighted the abundantly expressed CGRP as a crucial psychoactive mediator in a variety of neurobehavioral and psycho-affective conditions [40]. Thus, establishing the contribution of endogenous CSF CGRP to neuropsychological outcome and hrQoL in sSAH is rather promising.

### Excessive release of CGRP after sSAH in a neurobehavioral context

Our study proves an excessive hypersecretion of CGRP into CSF (mean level of 0.6 ng/ml) within the first 10 days after sSAH. A historic population [33] of 29 non-neurosurgical patients (15 women and 14 men; mean age 52.8 years) has provided insight into normal reference values of CSF CGRP. From these 29 patients, spinal CSF with a remarkably lower CGRP concentration (mean CGRP 0.09 ng/ml) was drawn during spinal anesthesia for minor orthopedic or urologic surgery [33]. In accordance with the psycho-behavioral literature, our study postulates a detrimental effect of CSF CGRP on psychological and physical health: Increased CSF AUC CGRP levels were significantly positively correlated to depression, anxiety, compulsive-obsessive syndrome, and the supplementary ISR items (which imply a variety of concomitant syndromes including problems with activities of daily living, sleep, concentration, flash backs, obliviousness, feelings of displacement and alienation, suicidality, appetite, and non-organic sexual dysfunction). A significantly negative correlation was established between high CSF AUC CGRP concentrations and the PCS score (covering Pfi, Rolph, Pain, and Ghp). At the supraspinal level, CGRP is broadly distributed like, for example, in the sensory and the trigeminal ganglia, the striatum, amygdala, pituitary gland, hypothalamus, medulla oblongata, and in the cortex [34, 37, 40]. The widespread presence of CGRP and its binding sites in the brain, eminently in limbic structures, indicates its potential involvement in a plethora of neurophysiological and neurobehavioral functions [40], like in depression [41, 42, 44], possibly in dementia [50], and in the pathophysiology of inflammatory and neuropathic pain [51–53]. Mathé and collaborators defined CGRP in lumbar CSF as a trait marker of major depression [44]. When administered intracerebroventricularly, intracerebrally, or intravenously in animal models, exogenous CGRP was found to potentiate fear-related behaviors [48], and it was attested a pivotal role in learning and consolidation of memory in passive avoidance tests [49], in locomotion, nociception, depression-like behaviors [43, 45–47], in anorexia [70], and in addiction [71–73]. Our multiple logistic regression analysis indicates a contribution of further clinical variables and, in addition, an interaction of the restrictions in most hrQoL domains.

Anxiety and depression [11, 17, 18] are among the most investigated realms of patient outcome after aSAH with a stable prevalence over the 18-month period after aSAH and an estimated frequency ranging from 27 to 54% and from 5 to 50% in aSAH survivors, respectively [9]. Likewise, somatoform disorders, especially pain syndromes like cephalgia, are a commonly reported and oftentimes a long-lasting symptom burden after sSAH, plausibly affecting Ghp, poorer performance in several cognitive domains, and reduced hrQoL after sSAH [9, 74]. In this context, the cerebral exposure to CGRP might reveal advanced insight and a potential therapeutic target in the future.

#### Release of CGRP after sSAH

The vasodilatatory CGRP [35] has been demonstrated to be excessively released into CSF [31, 33] and, to an even greater extent, into serum [31, 75] during the first 10 days [33, 75] after SAH. Endogenous CSF CGRP (upregulated on days 1 to 4 after sSAH) [33] and exogenously administered CGRP are postulated to be cerebroprotective and, thus, beneficial for functional outcome by preventing sSAH-induced CV and cerebral ischemia, respectively [28, 29]. The substantiated effects of CGRP on hemodynamics and, consecutively, on neurological outcome after sSAH contrast with the current findings, suggesting at least a contribution of CGRP to psychobehavioral dysregulation and reduced hrQoL.

The neuroanatomical circuitry involved in CGRP transmission and modulation remains to be clarified. Early observational studies on the cerebral circulation after experimental SAH and post mortem analyses after SAH confirmed a marked decrease in CGRP immunoreactivity in the perivascular nerve fibers (cf. references in [40]). Pathophysiologically, the neurotransmitter CGRP is supposed to be released from the perivascular nerve terminals, either induced by the blood in the subarachnoid space [76] or, possibly, caused by the direct affection or disruption of the neuropeptide-containing nerve fibers at the moment of aneurysm rupture and the subsequent inhibition of neuropeptide reuptake at the nerve-ending terminals. We propose that CSF CGRP may be dispersed with the circulating CSF from the basal cisterns into the ventricles where the neuropeptidergic concentrations are amenable to measurement. It is questionable whether CGRP in ventricular CSF reflects the proposed pathophysiological processes or whether it is rather mirroring other processes associated with critical illness.

It might further be speculated that the increase of endogenous CSF CGRP might be a consequence of altered CGRP synthesis and metabolism in certain brain regions and in CSF, respectively, anatomical localization of the ruptured aneurysm, or aneurysm treatment-induced mechanical manipulation of the parent vessel. In serum, peak concentrations of CGRP have been measured after rupture of aneurysms of the middle cerebral artery (MCA) [31, 75] and—regarding cerebrovascular manipulation—after endoluminal aneurysm treatment via coiling [75]. Our sSAH collective comprised a reference group with pSAH patients, characterized by conservative ICU management, to further illuminate the implication of the aneurysm-securing procedure on CGRP release and hrQoL outcome. Mean CGRP levels ranged lowest in the MS group. In line with subgroup analyses, MS patients demonstrated better short-term Pfi, experienced less pain, and more improvement in nutrition disorders than EV patients. As previously described, however, the treatment modality (MS vs. EV vs. pSAH) did not significantly affect CSF AUC CGRP levels, and overall hrQoL outcome did not differ between the MS and the EV group [21], either. We caveat the statement with the note that the small sample size of the current study is not capable for detecting any differences. Since 2002, the treatment modality-dependent outcome is controversially discussed [77]. The majority of authors disproved the hypothesis that clipped and coiled patients differ with respect to cognitive outcome, hrQoL, return to work, depression, anxiety, and sleep disturbances (cf. [9, 78] and references within), though.

It has to be highlighted that—in many ways—pSAH has to be considered a different disease than aSAH. Short-term complications are rare, and long-term outcome is excellent with respect to disability and death [6, 8]. aSAH implies a more aggressive clinical presentation, a more diffuse distribution of subarachnoid blood, a higher probability of complications, and a longer inpatient period with higher economical costs for health care systems. Yet, both, aSAH and pSAH patients, suffer from neuropsychological deficits and hrQoL restrictions after ictus [7, 9, 79]. Contrary to former assumptions, which attested pSAH patients a favorable prognosis [80, 81], more recent findings [7, 82, 83] indicated that pSAH might not be as benign as previously believed. On average 39 months after pSAH, survivors continued suffering from cephalgia, dizziness, fatigue, irritability, depression, obliviousness, mild cognitive deficits, and incapacity to resume their previous occupations [82, 83]. Long-term studies on potential cognitive and hrQoL sequelae after pSAH are demanded [8] and should separately address the CGRP effect on outcomes for pSAH and aSAH.

Investigations into the temporary dynamics of sSAH-induced CGRP secretion are scarce and limited to the short-term [84]. As our sSAH patients significantly improved in multiple physical and emotional hrQoL items within 6 months, it might be reasoned that CGRP concentrations decrease or even normalize over time. Hypothetically, extra- and intraluminal CGRP receptors may finally be saturated non-competitively with the peptide, and CSF levels decrease due to the depletion of the releasing terminal nerve endings. In turn, lower CSF CGRP concentrations or reuptake of ventricular CGRP may be beneficial to hrQoL and mental health. In 2013, our research group detected peak concentrations of CSF CGRP during the first 4 days after onset of sSAH, followed by a gradual decrease [33]. Nozaki et al. [85] found the most marked suppression of CGRP immunoreactivity during the 7th to 14th day with a recovery to normal levels by the 42nd day after artificial SAH. Congruently, our results suggest that at least within the first 10 days after sSAH, elevated CSF CGRP levels account for the sSAH-related psychological traumatization and reduced hrQoL. At the 6-month FU, self-reported hrQoL did no longer correlate with the initial CGRP values. Neuropsychological deficits, predominantly in hrQoL, cognition, depression, anxiety, mood, and fatigue [9–11, 17, 18, 86], are—consistently with our findings—most common within the first 3 months [87] but may persist as long as 24.5 years after ictus or even longer [60, 71, 18, 55, 1].

The pathophysiology of impaired neurobehavioral processing following sSAH remains elusive because of its complex, multifactorial character [22]. Various predictors of unfavorable neuropsychological outcome after aSAH (like the HH score and the GOS in our results section) have been proposed [5, 21]. In cognitively impaired good-grade sSAH patients without morphological changes in neuroimaging, it seems conceivable that the initial insult of the bleeding may result in a widespread derangement of peptide neurotransmitter secretion in the brain [68]. We caveat our findings with the note that our experimental, hypotheses-generating pilot study was not designed to conclusively establish whether pathologically increased neurotransmitter secretion, in particular excessively elevated CGRP levels in supratentorial CSF, induce restrictions in hrQoL and emotional health. However, a contribution seems feasible, given the fact that such associations exist in a myriad of psychiatric and neurobehavioral disorders. Thus, we argue that, in sSAH, CGRP acts as an elementary psychoactive mediator in higher integrated behavior.

#### Methodological considerations

Bounded by our strict selection criteria and our institutional neurovascular volume, our pilot study is notably limited by the small sample size. It may be speculated that the five excluded patients were incapable to complete the hrQoL assessment due to severe neuropsychological impairment, resulting in an underestimation of the true impairment rate. Then, the inclusion of pSAH patients as a control group implicates a certain collective heterogeneity because pSAH and aSAH have a different pathogenesis, clinical course, different rates of CV and delayed ischemic neurological deficits, and also different neuropsychological and hrQoL outcomes. Though pSAH patients are deemed equally burdened by neuropsychological deficits and reduced hrQoL [9], the outcome of aSAH patients is considered even worse. Therefore, future studies should separately address the CGRP effect on outcomes in both entities. Additionally, our study is severely biased by (1) the typical [78] overrepresentation of prognostically favorable good-grade sSAH patients, (2) the predominance of MCA aneurysms, which might have a significant confounding effect on outcomes, given very different potential perforator injuries and the extent of dissection required, and (3) the selection of patients with radiologically confirmed hydrocephalus. By nature, non-hydrocephalus patients are not amenable to CSF biomarker sampling. As a consequence, our data is not applicable to sSAH patients in general.

Multiple previous SAH investigations have used the SF-36, even in poor-grade SAH, showing an impact on all tested items of the SF-36 (cf. references in [12]). Both of our utilized measurement tools, the SF-36 and the ISR, were completed within 10 to 20 min and no effects of fatigue were apparent during the testing, neither at t_1_ nor at t_2_. However, we stress that in patients with a central nervous system disease, a confounding influence of fatigue [86] and/or cognitive impairment [88] cannot conclusively be excluded. Further research is necessary to develop assessments sensitive for the specific pattern of deficits in patients with intracranial aneurysms [5, 89]. Since statistically relevant correlations only occurred in the short term, a false-positive result has to be considered. Experiencing a traumatic and life-threatening event like the acute phase of an sSAH with consecutive ICU treatment expectedly bears the risk of low psychological and hrQoL scores. In concert with the supposed widespread derangement of neurotransmitters, CGRP, being part of an acute neurobiological response, is unsurprisingly high during this period. Conclusively, the established positive correlations have to be interpreted with caution. Our observational, correlative clinical pilot study prevents drawing final conclusions, establishing clear associations or implying causality.

Yet, our prospective, controlled study provides academically valuable data that offer new insight into the plausible interactions between CGRP in supratentorial CSF and psychopathology after good-grade sSAH as well as into the (to date still underreported) time course of hrQoL performance in the early stages of recovery. Standardized self-reported outcome measures are an important facet of a comprehensive hrQoL outcome assessment, the more since cognitive domain deficits are further complicated by reduced hrQoL, depression, anxiety, and sleep disturbances.

## Conclusion

Our study reveals the first insight into the potential capacity of endogenous CGRP as a predictive psychoactive biomarker in the ventricular CSF subacutely after the onset of sSAH and its potential contribution to neurobehavioral impairment and reduced hrQoL. In line with preclinical data and the psychiatric literature, the present data suggests that, after sSAH, increased CSF CGRP concentrations significantly adversely affect short-term psychological and physical health with respect to depression, anxiety, somatoform syndrome, compulsive-obsessive syndrome, the supplementary ISR items, general health perceptions, and the SF-36 physical component summary score. Of special note is the potential therapeutic dilemma as, on the one hand, it is highly conceivable that the psycho- and vasoactive neuropeptide CGRP is—at least in part—involved in the pathogenesis of reduced hrQoL after good-grade sSAH, whereas on the other hand, CGRP was certified a cerebroprotective role by counteracting sSAH-induced vasoconstriction and CV-related cerebral ischemia. Our interesting results justify further research on endogenous CGRP in CSF and plasma with a focus on the influencing factors of its release, the temporary dynamics, and the pathophysiological interactions with higher integrated neurobehavior.

## Data Availability

The data that support the findings of this study are available from the corresponding author upon reasonable request.

## References

[1] Lovelock CE, Rinkel GJ, Rothwell PM (2010). Time trends in outcome of subarachnoid hemorrhage: population-based study and systematic review. Neurology.

[2] Vergouwen MD, Jong-Tjien-Fa AV, Algra A, Rinkel GJ (2016). Time trends in causes of death after aneurysmal subarachnoid hemorrhage: a hospital-based study. Neurology.

[3] Long B, Koyfman A, Runyon MS (2017). Subarachnoid hemorrhage: updates in diagnosis and management. Emerg Med Clin North Am.

[4] Lantigua H, Ortega-Gutierrez S, Schmidt JM, Lee K, Badjatia N, Agarwal S, Claassen J, Connolly ES, Mayer SA (2015). Subarachnoid hemorrhage: who dies, and why?. Crit Care.

[5] Macdonald RL, Schweizer TA (2017). Spontaneous subarachnoid haemorrhage. Lancet.

[6] Coelho LG, Costa JM, Silva EI (2016). Non-aneurysmal spontaneous subarachnoid hemorrhage: perimesencephalic versus non-perimesencephalic. Rev Bras Ter Intensiva.

[7] Kapadia A, Schweizer TA, Spears J, Cusimano M, Macdonald RL (2014). Nonaneurysmal perimesencephalic subarachnoid hemorrhage: diagnosis, pathophysiology, clinical characteristics, and long-term outcome. World Neurosurg.

[8] Mensing LA, Vergouwen MDI, Laban KG, Ruigrok YM, Velthuis BK, Algra A, Rinkel GJE (2018). Perimesencephalic hemorrhage: a review of epidemiology, risk factors, presumed cause, clinical course, and outcome. Stroke.

[9] Al-Khindi T, Macdonald RL, Schweizer TA (2010). Cognitive and functional outcome after aneurysmal subarachnoid hemorrhage. Stroke.

[10] Haug Nordenmark T, Karic T, Roe C, Sorteberg W, Sorteberg A (2019) The post-aSAH syndrome: a self-reported cluster of symptoms in patients with aneurysmal subarachnoid hemorrhage. J Neurosurg:1–10. 10.3171/2019.1.JNS183168 10.3171/2019.1.JNS183168 31003212

[11] Persson HC, Tornbom M, Winso O, Sunnerhagen KS (2019). Symptoms and consequences of subarachnoid haemorrhage after 7 years. Acta Neurol Scand.

[12] Sonesson B, Kronvall E, Saveland H, Brandt L, Nilsson OG (2018). Long-term reintegration and quality of life in patients with subarachnoid hemorrhage and a good neurological outcome: findings after more than 20 years. J Neurosurg.

[13] van Gijn J, Rinkel GJ (2001). Subarachnoid haemorrhage: diagnosis, causes and management. Brain.

[14] Dey S, Kumar JK, Shukla D, Bhat D (2018). Neurological, neuropsychological, and functional outcome after good grade aneurysmal subarachnoid hemorrhage. Neurol India.

[15] Passier PE, Visser-Meily JM, van Zandvoort MJ, Post MW, Rinkel GJ, van Heugten C (2010). Prevalence and determinants of cognitive complaints after aneurysmal subarachnoid hemorrhage. Cerebrovasc Dis.

[16] Taufique Z, May T, Meyers E, Falo C, Mayer SA, Agarwal S, Park S, Connolly ES, Claassen J, Schmidt JM (2016). Predictors of poor quality of life 1 year after subarachnoid hemorrhage. Neurosurgery.

[17] Ackermark PY, Schepers VP, Post MW, Rinkel GJ, Passier PE, Visser-Meily JM (2017). Longitudinal course of depressive symptoms and anxiety after aneurysmal subarachnoid hemorrhage. Eur J Phys Rehabil Med.

[18] Tang WK, Wang L, Kwok Chu Wong G, Ungvari GS, Yasuno F, Tsoi KKF, Kim JS (2020). Depression after subarachnoid hemorrhage: a systematic review. J Stroke.

[19] Buunk AM, Spikman JM, Metzemaekers JDM, van Dijk JMC, Groen RJM (2019). Return to work after subarachnoid hemorrhage: the influence of cognitive deficits. PLoS One.

[20] Al Yassin A, Ouyang B, Temes R (2017). Depression and anxiety following aneurysmal subarachnoid hemorrhage are associated with higher six-month unemployment rates. J Neuropsychiatr Clin Neurosci.

[21] Brundl E, Schodel P, Bele S, Proescholdt M, Scheitzach J, Zeman F, Brawanski A, Schebesch KM (2017) Treatment of spontaneous subarachnoid hemorrhage and self-reported neuropsychological performance at 6 months - results of a prospective clinical pilot study on good-grade patients. Turk Neurosurg. 10.5137/1019-5149.JTN.21825-17.0 10.5137/1019-5149.JTN.21825-17.0 29204979

[22] Rinkel GJ, Algra A (2011). Long-term outcomes of patients with aneurysmal subarachnoid haemorrhage. Lancet Neurol.

[23] Stienen MN, Smoll NR, Weisshaupt R, Fandino J, Hildebrandt G, Studerus-Germann A, Schatlo B (2014). Delayed cerebral ischemia predicts neurocognitive impairment following aneurysmal subarachnoid hemorrhage. World Neurosurg.

[24] Brundl E, Proescholdt M, Schodel P, Bele S, Hohne J, Zeman F, Stoerr EM, Brawanski A, Schebesch KM (2018) Excessive release of endogenous neuropeptide Y into cerebrospinal fluid after treatment of spontaneous subarachnoid haemorrhage and its possible impact on self-reported neuropsychological performance - results of a prospective clinical pilot study on good-grade patients. Neurol Res:1–13. 10.1080/01616412.2018.1508547 10.1080/01616412.2018.1508547 30213237

[25] Heilig M, Widerlov E (1990). Neuropeptide Y: an overview of central distribution, functional aspects, and possible involvement in neuropsychiatric illnesses. Acta Psychiatr Scand.

[26] Pluta RM, Deka-Starosta A, Zauner A, Morgan JK, Muraszko KM, Oldfield EH (1992). Neuropeptide Y in the primate model of subarachnoid hemorrhage. J Neurosurg.

[27] Schebesch KM, Brawanski A, Bele S, Schodel P, Herbst A, Brundl E, Kagerbauer SM, Martin J, Lohmeier A, Stoerr EM, Proescholdt M (2013). Neuropeptide Y - an early biomarker for cerebral vasospasm after aneurysmal subarachnoid hemorrhage. Neurol Res.

[28] (1992) Effect of calcitonin-gene-related peptide in patients with delayed postoperative cerebral ischaemia after aneurysmal subarachnoid haemorrhage. European CGRP in Subarachnoid Haemorrhage Study Group. Lancet 339:831–834 1347857

[29] Johnston FG, Bell BA, Robertson IJ, Miller JD, Haliburn C, O'Shaughnessy D, Riddell AJ, O’Laoire SA (1990). Effect of calcitonin-gene-related peptide on postoperative neurological deficits after subarachnoid haemorrhage. Lancet.

[30] Juul R, Edvinsson L, Fredriksen TA, Ekman R, Brubakk AO, Gisvold SE (1990). Changes in the levels of neuropeptide Y-LI in the external jugular vein in connection with vasoconstriction following subarachnoid haemorrhage in man. Involvement of sympathetic neuropeptide Y in cerebral vasospasm. Acta Neurochir.

[31] Juul R, Hara H, Gisvold SE, Brubakk AO, Fredriksen TA, Waldemar G, Schmidt JF, Ekman R, Edvinsson L (1995). Alterations in perivascular dilatory neuropeptides (CGRP, SP, VIP) in the external jugular vein and in the cerebrospinal fluid following subarachnoid haemorrhage in man. Acta Neurochir.

[32] Kokkoris S, Andrews P, Webb DJ (2012). Role of calcitonin gene-related peptide in cerebral vasospasm, and as a therapeutic approach to subarachnoid hemorrhage. Front Endocrinol (Lausanne).

[33] Schebesch KM, Herbst A, Bele S, Schodel P, Brawanski A, Stoerr EM, Lohmeier A, Kagerbauer SM, Martin J, Proescholdt M (2013). Calcitonin-gene related peptide and cerebral vasospasm. J Clin Neurosci.

[34] Amara SG, Jonas V, Rosenfeld MG, Ong ES, Evans RM (1982). Alternative RNA processing in calcitonin gene expression generates mRNAs encoding different polypeptide products. Nature.

[35] Brain SD, Williams TJ, Tippins JR, Morris HR, MacIntyre I (1985). Calcitonin gene-related peptide is a potent vasodilator. Nature.

[36] Poyner DR (1992). Calcitonin gene-related peptide: multiple actions, multiple receptors. Pharmacol Ther.

[37] Rosenfeld MG, Mermod JJ, Amara SG, Swanson LW, Sawchenko PE, Rivier J, Vale WW, Evans RM (1983). Production of a novel neuropeptide encoded by the calcitonin gene via tissue-specific RNA processing. Nature.

[38] Hokfelt T, Arvidsson U, Ceccatelli S, Cortes R, Cullheim S, Dagerlind A, Johnson H, Orazzo C, Piehl F, Pieribone V (1992). Calcitonin gene-related peptide in the brain, spinal cord, and some peripheral systems. Ann N Y Acad Sci.

[39] McCulloch J, Uddman R, Kingman TA, Edvinsson L (1986). Calcitonin gene-related peptide: functional role in cerebrovascular regulation. Proc Natl Acad Sci U S A.

[40] van Rossum D, Hanisch UK, Quirion R (1997). Neuroanatomical localization, pharmacological characterization and functions of CGRP, related peptides and their receptors. Neurosci Biobehav Rev.

[41] Cizza G, Marques AH, Eskandari F, Christie IC, Torvik S, Silverman MN, Phillips TM, Sternberg EM, Group PS (2008). Elevated neuroimmune biomarkers in sweat patches and plasma of premenopausal women with major depressive disorder in remission: the POWER study. Biol Psychiatry.

[42] Hartman JM, Berger A, Baker K, Bolle J, Handel D, Mannes A, Pereira D, St Germain D, Ronsaville D, Sonbolian N, Torvik S, Calis KA, Phillips TM, Cizza G, Group POWERS (2006). Quality of life and pain in premenopausal women with major depressive disorder: the POWER Study. Health Qual Life Outcomes.

[43] Jiao J, Opal MD, Dulawa SC (2013). Gestational environment programs adult depression-like behavior through methylation of the calcitonin gene-related peptide gene. Mol Psychiatry.

[44] Mathe AA, Agren H, Lindstrom L, Theodorsson E (1994). Increased concentration of calcitonin gene-related peptide in cerebrospinal fluid of depressed patients. A possible trait marker of major depressive disorder. Neurosci Lett.

[45] Schorscher-Petcu A, Austin JS, Mogil JS, Quirion R (2009). Role of central calcitonin gene-related peptide (CGRP) in locomotor and anxiety- and depression-like behaviors in two mouse strains exhibiting a CGRP-dependent difference in thermal pain sensitivity. J Mol Neurosci.

[46] Shao B, Zhou YL, Wang H, Lin YS (2015). The role of calcitonin gene-related peptide in post-stroke depression in chronic mild stress-treated ischemic rats. Physiol Behav.

[47] Wortwein G, Husum H, Andersson W, Bolwig TG, Mathe AA (2006). Effects of maternal separation on neuropeptide Y and calcitonin gene-related peptide in “depressed” Flinders Sensitive Line rats: a study of gene-environment interactions. Prog Neuro-Psychopharmacol Biol Psychiatry.

[48] Sink KS, Chung A, Ressler KJ, Davis M, Walker DL (2013). Anxiogenic effects of CGRP within the BNST may be mediated by CRF acting at BNST CRFR1 receptors. Behav Brain Res.

[49] Kovacs A, Telegdy G (1995). Effects of CGRP on active avoidance behavior in rats. Physiol Behav.

[50] Mathe AA, Agren H, Wallin A, Blennow K (2002). Calcitonin gene-related peptide and calcitonin in the CSF of patients with dementia and depression: possible disease markers. Prog Neuro-Psychopharmacol Biol Psychiatry.

[51] Benarroch EE (2011). CGRP: sensory neuropeptide with multiple neurologic implications. Neurology.

[52] Benemei S, Nicoletti P, Capone JG, Geppetti P (2009). CGRP receptors in the control of pain and inflammation. Curr Opin Pharmacol.

[53] Yu LC, Hou JF, Fu FH, Zhang YX (2009). Roles of calcitonin gene-related peptide and its receptors in pain-related behavioral responses in the central nervous system. Neurosci Biobehav Rev.

[54] Close LN, Eftekhari S, Wang M, Charles AC, Russo AF (2018) Cortical spreading depression as a site of origin for migraine: role of CGRP. Cephalalgia:333102418774299. 10.1177/0333102418774299 10.1177/0333102418774299 PMC7007998 29695168

[55] Edvinsson L, Jansen I, Cunha e Sa M, Gulbenkian S (1994). Demonstration of neuropeptide containing nerves and vasomotor responses to perivascular peptides in human cerebral arteries. Cephalalgia.

[56] Ho TW, Edvinsson L, Goadsby PJ (2010). CGRP and its receptors provide new insights into migraine pathophysiology. Nat Rev Neurol.

[57] Hunt WE, Hess RM (1968). Surgical risk as related to time of intervention in the repair of intracranial aneurysms. J Neurosurg.

[58] Teasdale GM, Drake CG, Hunt W, Kassell N, Sano K, Pertuiset B, De Villiers JC (1988). A universal subarachnoid hemorrhage scale: report of a committee of the World Federation of Neurosurgical Societies. J Neurol Neurosurg Psychiatry.

[59] Jennett B, Bond M (1975). Assessment of outcome after severe brain damage. Lancet.

[60] Rankin J (1957). Cerebral vascular accidents in patients over the age of 60. II. Prognosis. Scott Med J.

[61] Kagerbauer SM, Kemptner DM, Schepp CP, Bele S, Rothorl RD, Brawanski AT, Schebesch KM (2010). Elevated premorbid body mass index is not associated with poor neurological outcome in the subacute state after aneurysmal subarachnoid hemorrhage. Cent Eur Neurosurg.

[62] Woitzik J, Dreier JP, Hecht N, Fiss I, Sandow N, Major S, Winkler M, Dahlem YA, Manville J, Diepers M, Muench E, Kasuya H, Schmiedek P, Vajkoczy P, group Cs (2012). Delayed cerebral ischemia and spreading depolarization in absence of angiographic vasospasm after subarachnoid hemorrhage. J Cereb Blood Flow Metab.

[63] Brundl E, Bohm C, Lurding R, Schodel P, Bele S, Hochreiter A, Scheitzach J, Zeman F, Brawanski A, Schebesch KM (2016). Treatment of unruptured intracranial aneurysms and cognitive performance: preliminary results of a prospective clinical trial. World Neurosurg.

[64] Bullinger M (1995). German translation and psychometric testing of the SF-36 Health Survey: preliminary results from the IQOLA Project. International Quality of Life Assessment. Soc Sci Med.

[65] Tritt K, von Heymann F, Zaudig M, Zacharias I, Sollner W, Loew T (2008). Development of the “ICD-10-Symptom-Rating” (ISR) questionnaire. Z Psychosom Med Psychother.

[66] Ware JE, Snow KK, Kosinski M, Gandek B (1993). SF-36 Health Survey: manual and interpretation guide.

[67] Joswig H, Korte W, Fruh S, Epprecht L, Hildebrandt G, Fournier JY, Stienen MN (2018). Neurodegenerative cerebrospinal fluid biomarkers tau and amyloid beta predict functional, quality of life, and neuropsychological outcomes after aneurysmal subarachnoid hemorrhage. Neurosurg Rev.

[68] Uski TK, Lilja A, Saveland H, Ekman R, Sonesson B, Brandt L (2000). Cognitive functioning and cerebrospinal fluid concentrations of neuropeptides for patients with good neurological outcomes after aneurysmal subarachnoid hemorrhage. Neurosurgery.

[69] Uddman R, Edvinsson L, Ekblad E, Hakanson R, Sundler F (1986). Calcitonin gene-related peptide (CGRP): perivascular distribution and vasodilatory effects. Regul Pept.

[70] Dhillo WS, Small CJ, Jethwa PH, Russell SH, Gardiner JV, Bewick GA, Seth A, Murphy KG, Ghatei MA, Bloom SR (2003). Paraventricular nucleus administration of calcitonin gene-related peptide inhibits food intake and stimulates the hypothalamo-pituitary-adrenal axis. Endocrinology.

[71] Ehlers CL, Somes C, Li TK, Lumeng L, Hwang BH, Jimenez P, Mathe AA (1999). Calcitonin gene-related peptide (CGRP) levels and alcohol. Int J Neuropsychopharmacol.

[72] Salmon AM, Damaj MI, Marubio LM, Epping-Jordan MP, Merlo-Pich E, Changeux JP (2001). Altered neuroadaptation in opiate dependence and neurogenic inflammatory nociception in alpha CGRP-deficient mice. Nat Neurosci.

[73] Zhou X, Li JJ, Yu LC (2003). Plastic changes of calcitonin gene-related peptide in morphine tolerance: behavioral and immunohistochemical study in rats. J Neurosci Res.

[74] Huckhagel T, Klinger R, Schmidt NO, Regelsberger J, Westphal M, Czorlich P (2020). The burden of headache following aneurysmal subarachnoid hemorrhage: a prospective single-center cross-sectional analysis. Acta Neurochir.

[75] Schebesch KM, Bründl E, Hochreiter A, Scheitzach J, Bele S, Herbst A, Brawanski A, Proescholdt M, Lohmeier A, Stoerr EM, Schoedel P (2014). Calcitonin gene-related peptide in serum after spontaneous subarachnoid hemorrhage. American Journal of Neuroscience.

[76] Edvinsson L, Delgado-Zygmunt T, Ekman R, Jansen I, Svendgaard NA, Uddman R (1990). Involvement of perivascular sensory fibers in the pathophysiology of cerebral vasospasm following subarachnoid hemorrhage. J Cereb Blood Flow Metab.

[77] Scott RB, Eccles F, Molyneux AJ, Kerr RS, Rothwell PM, Carpenter K (2010). Improved cognitive outcomes with endovascular coiling of ruptured intracranial aneurysms: neuropsychological outcomes from the International Subarachnoid Aneurysm Trial (ISAT). Stroke.

[78] Egeto P, Loch Macdonald R, Ornstein TJ, Schweizer TA (2017). Neuropsychological function after endovascular and neurosurgical treatment of subarachnoid hemorrhage: a systematic review and meta-analysis. J Neurosurg.

[79] Boerboom W, Heijenbrok-Kal MH, Khajeh L, van Kooten F, Ribbers GM (2014). Differences in cognitive and emotional outcomes between patients with perimesencephalic and aneurysmal subarachnoid haemorrhage. J Rehabil Med.

[80] Brilstra EH, Hop JW, Rinkel GJ (1997). Quality of life after perimesencephalic haemorrhage. J Neurol Neurosurg Psychiatry.

[81] Rinkel GJ, Wijdicks EF, Vermeulen M, Hageman LM, Tans JT, van Gijn J (1990). Outcome in perimesencephalic (nonaneurysmal) subarachnoid hemorrhage: a follow-up study in 37 patients. Neurology.

[82] Madureira S, Canhao P, Guerreiro M, Ferro JM (2000). Cognitive and emotional consequences of perimesencephalic subarachnoid hemorrhage. J Neurol.

[83] Marquardt G, Niebauer T, Schick U, Lorenz R (2000). Long term follow up after perimesencephalic subarachnoid haemorrhage. J Neurol Neurosurg Psychiatry.

[84] Tran Dinh YR, Debdi M, Couraud JY, Creminon C, Seylaz J, Sercombe R (1994). Time course of variations in rabbit cerebrospinal fluid levels of calcitonin gene-related peptide- and substance P-like immunoreactivity in experimental subarachnoid hemorrhage. Stroke.

[85] Nozaki K, Kikuchi H, Mizuno N (1989). Changes of calcitonin gene-related peptide-like immunoreactivity in cerebrovascular nerve fibers in the dog after experimentally produced subarachnoid hemorrhage. Neurosci Lett.

[86] Kutlubaev MA, Barugh AJ, Mead GE (2012). Fatigue after subarachnoid haemorrhage: a systematic review. J Psychosom Res.

[87] Powell J, Kitchen N, Heslin J, Greenwood R (2002). Psychosocial outcomes at three and nine months after good neurological recovery from aneurysmal subarachnoid haemorrhage: predictors and prognosis. J Neurol Neurosurg Psychiatry.

[88] Wong GK, Lam S, Ngai K, Wong A, Mok V, Poon WS, Cognitive Dysfunction after Aneurysmal Subarachnoid Haemorrhage I (2012). Evaluation of cognitive impairment by the Montreal cognitive assessment in patients with aneurysmal subarachnoid haemorrhage: prevalence, risk factors and correlations with 3 month outcomes. J Neurol Neurosurg Psychiatry.

[89] Zaki Ghali MG, Srinivasan VM, Wagner K, Rao C, Chen SR, Johnson JN, Kan P (2018). Cognitive sequelae of unruptured and ruptured intracranial aneurysms and their treatment: modalities for neuropsychological assessment. World Neurosurg.

